# Effect of Group Positive Psychotherapy on Improvement of Life Satisfaction and The Quality of Life in Infertile Woman

**DOI:** 10.22074/ijfs.2016.4775

**Published:** 2016-04-05

**Authors:** Seyed Teymur Seyedi Asl, Kheirollah Sadeghi, Mitra Bakhtiari, Seyed Mojtaba Ahmadi, Alireza Nazari Anamagh, Tayebeh Khayatan

**Affiliations:** 1 Department of Psychology, Faculty of Education and Psychology, Mohaghegh Ardabili University, Ardabil, Iran; 2 Department of Psychology, Faculty of Medicine, Kermanshah University of Medical Sciences, Kermanshah, Iran; 3 Fertility and Infertility Research Center, Faculty of Medicine, Kermanshah University of Medical Sciences, Kermanshah, Iran; 4 Department of Humanities and Social Sciences, Science and Research Branch of Islamic Azad University, Tehran, Iran; 5 Department of Psychology, University of Social Welfare and Rehabilitation Sciences, Tehran, Iran

**Keywords:** Psychotherapy, Female Infertility, Quality of Life, Depression

## Abstract

**Background:**

Positive psychotherapy is one of the new approaches in psychology which is innovated for treating psychological disorders and enhancing positive emotions. The aim of this study is to investigate the effectiveness of the group positive psychotherapy on elevation of life satisfaction and quality of life in infertile women.

**Materials and Methods:**

In a randomized trial study, Beck Depression Inventory II (BDI-II) and clinical interview were used in a pre-test post-test control group design. After analyzing the result of the questionnaire, 36 infertile women who showed signs of mild to moderate depression were randomly placed into two following groups: control (n=18) and intervention (n=18). Before the treatment, the members of both groups answered BDI-II, Satisfaction With Life Scale (SWLS) and 12 item Short Form Health Survey (SF-12). The intervention group received six sessions of group positive psychotherapy, but the treatment of the control group began six weeks after the intervention group.

**Results:**

The results showed that the life satisfaction scores of the intervention group were significantly elevated from 22.66 in pre-test to 26.13 in post-test (P<0.001), while this improvement was not significant in the control group (P=0.405). The difference between life satisfaction scores of the intervention and the control groups was also significant (F=8.92, P=0.006). However, no significant change in the quality of life level of the intervention and control groups was observed (P=0.136).

**Conclusion:**

Thus it can be deduced from the findings that this treatment method could be introduced as solution to increase the life satisfaction in infertile women, but not as a treatment for elevating their quality of life (Registration Number: IRCT2013042810063N3).

## Introduction

As the science has advanced, many of the once untreatable illnesses have now become treatable. However, there are certain problems which could still inflict a great deal of stress on people. Infertility is a reproductive system disease which is defined as inability to achieve clinical pregnancy 12 months after having sexual intercourse without any prophylactic device (1). It has been shown that the prevalence of lifetime infertility is between 6.6 to 26.4% worldwide, of which 9% showed infertility duration of 12 months. In 2007, 72 million women suffered from infertility worldwide (2). Also in another study (3), it has been shown that 11.2 to 14.1% of Iranian couples experienced one or another form of subfertility. Infertility is also considered as one of those diseases leading to mental health disorders (4). The high prevalence of infertility, therefore, necessitates the treatment for mental health stress of these couples. Approximately, one third of the infertility is associated with factors which are related to women, one third is related to men and one third is related to both men and women (3). Despite all this, infertility is still considered as woman’s problem, especially in the context of developing countries (4). For example, in Iranian culture, the social implications of divorce, remarriage and separation causes severe mental stress for infertile women (5). The infertility is suggested to be associated with bio-psychosocial crisis (4), meaning stress contributing to many of the psychological disorders are the side-effects of infertility (6). Therefore, along with treatment of infertility, the individuals’ mental health and social problems must be paid attention as well. Several studies have reported that sexual dysfunction (7), eating disorders (8), depression disorder (9,11), and psychiatric disorders (12,14) are more common in infertile women as competed to the women with a healthy reproductive system. Furthermore sexual desire and arousal are lower in infertile women (15). 

Another common problem of the infertile women is the decreased levels of life satisfaction and quality of life. Life satisfaction is a cognitive and judgmental process that is based on comparison of the individual’s conditions with what is considered as a proper standard (16). In a study by Callan and Hennessey (17), they found that the infertile women were less satisfied with their lives than the fertile ones. In addition quality of life as a multidimensional factor includes cognitive, behavioral capacities, emotional well-being and capabilities which are necessary for performing family, social and vocational roles (18). Another research showed that health-related quality of life and sexual function are significantly lower in the women with primary infertility (19). Also in a research, it has found that quality of life is lower in the infertile women than the men (20). 

Many different methods have been developed for treating the psychological problems of the infertile individuals. For example, interpersonal psychotherapy (IPT) (21), cognitive behavioral therapy (CBT), supportive psychotherapy (9,22), as well as emotion-focused and problem-focused coping strategies (23) have been known as a few of the useful methods. However, positive psychology is a new approach which emphasizes on the individuals’ strengths that includes the scientific study of positive emotions, positive individual traits and positive institutions (24). Positive psychology as an intervention technique is applied to promote positive experiences, positive behaviors or positive cognitions (25). This treatment method has been shown to be effective due to the following factors: helping those who do not respond positively to drug therapy, cost effective, taking a short period of time to improve positive mood, no stigmatization and no negative side-effects (26). Some researchers have shown that since positive psychotherapy and interventions were effective on happiness and depression, it is likely to influence quality of life and life satisfaction (24). Since no research has been conducted with regard to this issue in the infertile women, the aim of this research is, therefore, to study the effectiveness of the group positive psychotherapy on elevation of the life satisfaction and the quality of life in infertile women. 

## Materials and Methods

### Participants and procedure

This randomized trial study was approved by the Ethics Committee of the Kermanshah University of Medical Sciences, Kermanshah, Iran. During April and June 2013, the women paying a visit to Motazedi Infertility Treatment Center, Kermanshah, Iran, were selected using the convenience sampling method and asked to answer the Beck Depression Inventory II (BDI-II). All the participants were signed an informed consent before entering the study. A total of 121 individuals answered the questionnaire, of whom 115 fully completed the form. Then, a clinical psychologist conducted a diagnostic interview with those who showed mild to moderate symptoms of depression using the Diagnostic and Statistical Manual of Mental Disorders, Fourth Edition, Text Revision (DSMIV-TR). Ultimately 49 people were found to meet the criteria to be included in the groups, of whom only 40 people were accepted to participate in the experiment. Out of these 40 individuals (four patients due to infertility treatment were dropped from the study), 36 people were randomly placed into two groups of intervention (n=18) and control (n=18) in a pre-test post-test control group design. The intervention group was divided into three subgroups of six individuals who received positive psychotherapy. However, the treatment of those in the control group was delayed until the end of the experiment. Before the treatment began, the participants in both groups were asked to answer the questions in the questionnaire regarding the life satisfaction and the quality of life. 

**Fig.1:**
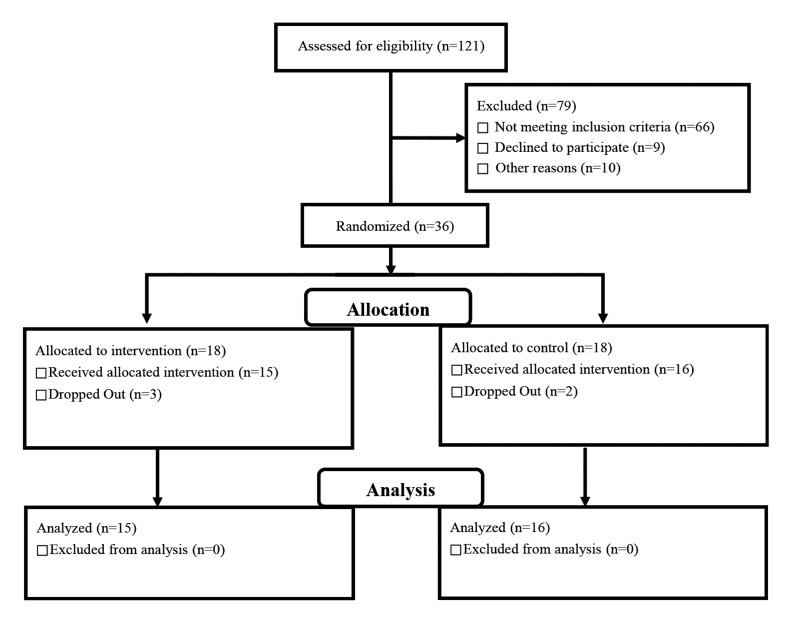
Graphic representation of participant flow.

The inclusion criteria in this study were as follows: i. Filling out the BDI-II, ii. Diagnosis of major depressive disorder (MDD) based on the major depression criteria mentioned in DSM-IV-TR criteria, iii. Not receiving psychiatric medication or any other form of psychotherapy treatment, and iv. Not having any other form of psychiatric disorder. The exclusion criteria in this study were as follows: i. Presence of physical problems interrupting the treatment process and ii. Presence of depression becoming more severe during the treatment period (in the event of intensified depression, the individuals from both groups were referred to a colleague who was a trained psychiatrist). Ultimately three people from the intervention group and two people from the control group were dropped out of the treatment process (Fig .1). 

### Instruments

#### Beck Depression Inventory II

The BDI-II measuring the symptoms for a two week-period contains 21 items. Each item is scored from 0 to 3 and total scores range from 0 to 63 that is interpreted as 0 to 13 for lowest depression, 14 to 19 for mild depression, 20 to 28 for moderate depression and 29 to 63 for severe depression (27). This questionnaire has copies in countries such as Japan (28) and Brazil (29). The Persian version of the questionnaire was evaluated with alpha value of 0.87 and test-retest reliability of 0.74 (30). 

### Satisfaction With Life Scale 

This scale was designed by Diener et al. (16) 

that is a very commonly used tool in researches on subjective-wellbeing (SWB) issues consisting of 5 items. Each item uses a Likert scale from 0 to 7 (31). In Iranian version, the reliability of Satisfaction With Life Scale (SWLS) is 0.83 when using the Cronbach Alpha and 0.69 when using the test-retest method. The structure reliability of this test is reported suitable using two other questionnaires (32). 

### 12-item Short Form Health Survey

The 12-item quality of life questionnaire is the shortened version of the 36-item Short Form Health Survey (SF-36) (33). This questionnaire consists of eight sub-scales. Since there are a few questions, only the overall score of the person was used in this study. A research conducted on 5586 people in Tehran determined the validity (Cronbach’s alpha= 0.72) and the reliability (with factor analysis) of this questionnaire, suggesting the questionnaire is suitable for the Iranian people both in terms of reliability and validity (34). 

### Intervention

The participants in this study were randomly placed into two groups of intervention and control. The treatment of the control group was delayed by six weeks, but the intervention group participated in a six-week group positive psychotherapy. This intervention was formulated and studied at the University of Pennsylvania in 2009 by Parks-Sheiner (35). Interventions were performed in a hospital room. Each session lasting an hour and half was held in a group therapy format. In each meeting, there were tasks that were completed by the participants for the following meeting. The meetings included the following six positive exercises: i. Using your strengths, ii. Gratitude visit, iii. A active-constructive response, iv. Counting blessings, v. Savoring, and vi. Biography (Table 1). The treatment sessions were carried out by a master degree student in clinical psychology program who had received training on positive psychotherapy at the Psychology and Counseling Organization of I.R. Iran. Moreover the intervention sessions were supervised by a tenuretrack professor of clinical psychology at Kermanshah University of Medical Sciences. 

**Table 1:** The definition of positive therapy and its sessions


Session	Content

Session one	Opening and positive introductions
	Preview next session and describe homework: value in action (VIA)/using your strengths
	Homework: take VIA strengths assessment to find out how to use one of your strengths every day
Session two	Discuss homework: using your strengths
	Preview next session and describe homework: gratitude
	Homework: write and deliver a gratitude letter
Session three	Discuss homework: gratitude letter
	Preview next session and describe homework: active-constructive responses
	Homework: make an active-constructive responses in social interactions
Session four	Discuss homework: active-constructive responses
	Preview next session and describe homework: blessings
	Homework: each night before bed, write down three good things that happened.
Session five	Discuss homework: blessings
	Preview next session and describe homework: savoring/biography
	Homework: pick one thing you usually rush through and take the time to savor it. Write a short essay (~1 page) detailing the characteristics and accomplishments that you hope to be remembered for and consider how much time you dedicated to pursue these Goals.
Session six	Discuss homework: biography and savoring
	Closing/maintenance
	Homework: pick at least one exercise and try to integrate it into your everyday life


### Statistical analysis

The independent-samples t test and Chi-square were used to compare the demographic information and the pre-test depression scores between two groups. In addition the paired-samples t test and analysis of covariance (ANCOVA) were applied to study the differences between the scores of life satisfaction and quality of life, pre-tests and post-tests, of both groups. 

## Results

The demographic characteristics and pre-test depression scores of both groups are shown in Table 2, indicating there are no significant differences regarding these variables between two groups. 

The results showed that life satisfaction significantly improved in the intervention group when comparing the results of the post-test with those of the pre-test (P<0.001), while this improvement was not significant in the control group (P=0.405). Moreover the quality of life showed no improvement in both groups when comparing the results of the post-test with those of the pretest (Table 3). 

Another finding of this study showed that life satisfaction displayed a significant increase in the intervention group in comparison to the control one (P=0.006), but there is no significant difference regarding this variable between two groups (Table 4). 

**Table 2:** Comparison of demographic characteristics and the pre-test depression scores between the control and intervention groups


Variables	Control group	Intervention group	Total sample	t or χ2	P value
	Mean (SD) n (%)	Mean (SD) n (%)	Mean (SD) n (%)		

Age	29.25 (5.65)	32.33 (4.82)	30.49 (5.68)	t=1.62	0.11
Husband,s age	34.63 (6.52)	37.60 (8.36)	35.11 (6.38)	t=1.10	0.27
Length of marriage (Y)	7.63 (5.89)	7.07 (5.65)	7.75 (5.36)	t=0.27	0.79
Infertility duration (Y)	3.93 (3.13)	5.27 (5.37)	4.45 (4.04)	t=0.83	0.41
BDI-II	21.87 (5.45)	20.87 (5.44)	19.43 (11.97)	t=0.51	0.61
Education					
Pre-high school	4 (25.0)	5 (33.3)	27 (23.5)		
High school	7 (43.8)	5 (33.3)	53 (46.1)	χ2=0.41	0.81
Higher education	5 (31.2)	5 (33.3)	35 (30.4)		
Sum	16	15	115		


BDI-II; Beck Depression Inventory-II, t; Independent t test and χ2; Chi-squared.

**Table 3:** Investigation of the differences of the variables between the pre-test and the post-test results of control and the intervention groups using paired-samples t test


Measurement	Group	Pre test		Post test		t	P value
		Mean	SD	Mean	SD		

SWLS	Intervention	22.66	4.48	26.13	4.10	5.56	P<0.001
	Control	21.06	4.73	21.68	5.79	0.086	0.405
SF-12	Intervention	31.00	5.02	33.53	4.83	1.96	0.069	
	Control	30.87	4.42	31.06	5.02	0.15	0.876


SWLS; Satisfaction With Life Scale, SF-12; 12-item Short Form Health Survey and t; Independent t test.

**Table 4:** Comparison of the differences between the control and the intervention groups after controlling pre-test using ANCOVA


	Source	SS	df	MS	F	P value	Eta-squared	Observedpower

SWLS	Pre-test	532.99	1	532.99	72.38	0.001>	0.72	
	Group	65.67	1	65.67	8.92	0.006	0.24	0.82
	Error	206.17	28	7.36				
	Total	18509.0	31					
SF-12	Pre-test	174.42	1	174.42	9.18	0.005	0.25	
	group	44.81	1	44.81	2.36	0.136	0.07	0.31
	Error	532.25	28	19.01				
	Total	753.93	31					


ANCOVA; Analysis of covariance, SWLS; Satisfaction With Life Scale, SF-12; 12-item Short Form Health Survey, SS; Sum of squares, df; Degree of freedom, MS; Mean squares and F; Function.

## Discussion

Infertility is a physical illness caused by several physical and emotional factors. A number of different approaches for infertility treatment have led to many psychological treatment methods. The first finding of this study showed that there was a significant increase in life satisfaction in the intervention group as compared to the control group. This finding is in line with the results of the meta-analysis of Sin and Lyubomirsky (25). In another study (36), 55 students were placed into two groups, an intervention group of 28 and a waiting list of 27. The intervention group underwent a 10 week-long Wellness Promotion Intervention. The life satisfaction of these students showed significant improvement in the post-test, whereas the well-being of the students in the waiting list showed a decline, even though this decline was not significant (36). Life satisfaction could be considered as a cognitive and judgmental process which is based on comparing the individual’s condition with what is considered as a proper standard (16). Lower life satisfaction in infertile women is verified in the previous study (17) that is more likely to be due to the fact that the infertile women find their conditions hopeless. Positive psychological interventions are certain treatment methods cultivating positive emotions, positive behaviors and positive cognitions (25). Considering the fact that these treatment methods emphasize on positive emotions and strengths, they both treat depression and enhance life satisfaction. For example, counting the blessings teaches the infertile women that even though they are deprived of having a blessing as important as having a child, they have thousands of other blessings in their lives which they should be thankful for.

Another finding of this study was that the quality of life showed no significant improvement both in the intervention and the control group. Since this study was the first of its kind, it had some limitations. The first limitation was the short duration of the treatment period. Although six weeks of the treatment saved time and expenses, it may not be enough time for some participants to see a positive result. It is recommended that the results of this short-term treatment to be compared with those of long-term treatments, so the best intervention method may be determined for the infertile women. The second limitation was related to the measurement tools. Due to limited number of questions regarding the quality of life factor, this was hard for the researcher to identify and separate the areas where the treatment was effective or ineffective. Moreover, life satisfaction in this study was measured using a five-question questionnaire, which may not be suitable to study the effectiveness of the treatment. It is recommended that in the future researches, a different questionnaire to be used. The third limitation in the study was that the sample participants were limited to the infertile women who were selected only from the residents of one city in Iran. Therefore, the results of this study may not apply to infertile women with different ethnicities. Finally this treatment method relies mostly on practice. However, those patients who were used to the structured sessions may not benefit from it.

## Conclusion

This study showed that group positive psychotherapy could be beneficial in elevating life satisfaction in infertile women. Considering the low cost of positive psychotherapy due to a less number of the sessions and easy method, this treatment could be used comprehensively in all the infertility treatment centers. The results of the study revealed that, for elevation of the quality of life in infertile women, other interventions must be used. However, more research must be carried out and the limitations of this study must be considered.
